# Radiocontrast Medium-Induced Kounis Syndrome in a Dialysis Patient: A Case Report

**DOI:** 10.7759/cureus.60014

**Published:** 2024-05-09

**Authors:** Genki Nakamura, Masayuki Ozaki, Yuma Yasuda, Takuya Yoshida, Takuya Inoue

**Affiliations:** 1 Emergency and Critical Care Medicine, Komaki City Hospital, Komaki, JPN

**Keywords:** radiocontrast medium, coronary angiography, hemodialysis, adrenaline, kounis syndrome

## Abstract

Kounis syndrome is defined as the concurrence of acute coronary syndrome and a condition related to mast cell activation, including anaphylaxis and anaphylactoid. A 58-year-old male hemodialysis patient underwent enhanced computed tomography (CT) using the radiocontrast medium, iopamidol for investigation of a kidney tumor. Two minutes after the administration of iopamidol, he developed respiratory symptoms and chest pain. Five minutes after that, disturbed consciousness and low blood pressure were observed. On the other hand, he did not demonstrate urticaria and swelling of the skin. A 12-lead electrocardiogram (ECG) and echocardiogram suggested the presence of cardiac ischemia. Therefore, he was diagnosed with Kounis syndrome caused by radiocontrast media. Eighteen minutes after this, he received an intramuscular injection of adrenaline (0.3 mg), and his vital signs stabilized and his ECG, echocardiogram, and symptoms improved. Without undergoing emergency coronary angiography (CAG), he was hospitalized and closely monitored. The next day, his symptoms had not worsened, and he underwent hemodialysis at his local hospital. The allergen radiocontrast media could be injurious and not sufficiently excreted if administrated for patients on weekly hemodialysis with radiocontrast medium-induced Kounis syndrome manifesting; hence, indication for emergency CAG in radiocontrast medium-induced Kounis syndrome should be cautiously evaluated by close observation.

## Introduction

Kounis syndrome is defined as the concurrence of acute coronary syndrome and conditions related to mast cell activation, including anaphylaxis and anaphylactoid reactions, which are reportedly caused by inflammatory mediators released during mast cell activation [1].

The clinical symptoms of Kounis syndrome include chest discomfort, dyspnoea, faintness, nausea, pruritus, and urticaria, and they are accompanied by signs such as hypotension, diaphoresis, pallor, and bradycardia. There are also ECG findings indicating myocardial ischemia, arrhythmias, and conduction defects [2]. This syndrome has been reported in conjunction with bee stings, antibiotics, and other causes [3-5]. Some case reports have described patients undergoing renal replacement treatment who have developed Kounis syndrome related to the hemodialysis membranes [6] or anticoagulants [7] used during hemodialysis. However, to the best of our knowledge, the syndrome has never been reported as caused by radiocontrast media in a patient undergoing hemodialysis.

Although radiocontrast medium-induced Kounis syndrome has been reported [8], this is the first reported case in a dialysis patient. Furthermore, this report is important in contributing to the understanding of radiocontrast medium-induced Kounis syndrome in dialysis patients, because for these patients the allergen, radiocontrast media, administrated in CAG needed for the treatment to be determined, could be harmful and not adequately removed from their body without getting hemodialysis.

This article was previously presented as a poster at the 2020 Japanese Association for Acute Medicine (JAAM) Annual Meeting on November 18, 2020.

## Case presentation

A 58-year-old man, with no risk factors for ischemic heart disease other than being a former smoker, receiving weekly hemodialysis for chronic kidney disease, underwent enhanced CT for investigation of a kidney tumor. Two minutes after the administration of the radiocontrast medium, iopamidol, during the CT, he developed respiratory symptoms (cough and dyspnea) and chest pain. Another two minutes passed, and disturbed consciousness was observed. Five minutes after that, he was transported to our emergency department (ED).

**Figure 1:**
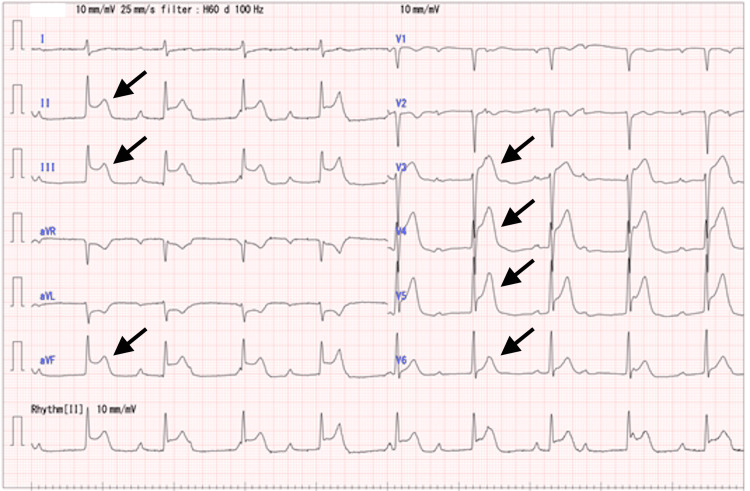
An electrocardiogram taken at the time of the patient’s admission to our emergency room. A complete atrioventricular block; ST elevation at II, III, aVF, V3-6; and ST depression at aVL were observed.

Upon arrival, his Glasgow Coma Scale score was E1V3M5, blood pressure was 55/31 mmHg, heart rate was 60 beats/minute, respiratory rate was 16 breaths/minute, body temperature was 36.3°C, and oxygen saturation was 90% while receiving 10 L/min of O2 via a face mask. The patient complained of chest pain and dyspnea, but there was no evidence of upper or lower airway stenosis or abnormalities of the skin, mucous membranes, or abdomen. Therefore, he was diagnosed with severe anaphylaxis due to the radiocontrast medium. Nine minutes after that, his ECG showed a complete atrioventricular block; ST elevation at II, III, aVF, V3-6; and ST depression at aVL (Figure 1) and echocardiogram displayed asynergy in the lateral wall and a part of the septum. His chest pain and ECG and echocardiogram findings also indicated acute coronary syndrome. To summarize his clinical features observed, after the administration, he had respiratory symptoms, low oxygen saturation, altered consciousness, hypotension, chest pain, and the ECG and echocardiogram findings indicative of acute coronary syndrome. Therefore, he was diagnosed with Kounis syndrome caused by radiocontrast media. 

Eighteen minutes after that, emergent anaphylaxis treatment was started which included an intramuscular injection of adrenaline (0.3 mg). Three minutes after the adrenaline, his ECG and echocardiogram readings and symptoms immediately improved. Another six minutes passed, and his blood pressure increased to 88/45 mmHg. We consulted with cardiologists regarding his myocardial ischemia and discussed the need for emergent CAG. Due to the patient’s rapid improvements (ECG and echocardiogram readings and symptoms), the risk of using radiocontrast media, the allergen, associated with CAG was determined to be higher than for hospitalization with careful observation.

**Figure 2:**
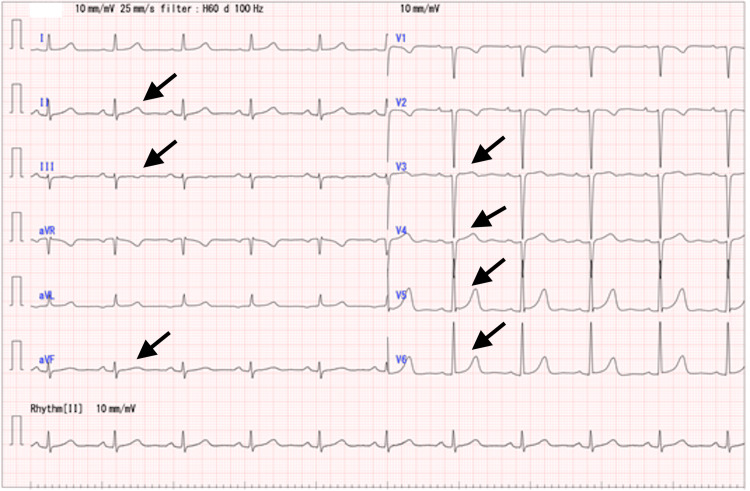
An electrocardiogram taken from the same patient on the following day showed normalization of atrioventricular block, ST elevation at II, III, aVF, V3-6, and ST depression at aVL

Following admission to a high-care unit, the patient received hydrocortisone (8 mg/kg) and chlorpheniramine maleate (5 mg). His ECG, chest pain, symptoms of anaphylaxis, and blood test (creatine kinase) were monitored. The next day, the patient did not demonstrate abnormal ECG readings (Figure 2), an increase of creatine kinase, or anaphylaxis symptoms, so progressing myocardial ischemia was not suspected; therefore, he was discharged and underwent hemodialysis at his local hospital, and he did not need follow-up monitoring or any further investigations.

## Discussion

Adrenaline is the first-line drug for treating anaphylaxis. A review presents that the large coronary arteries have a larger percentage of alpha receptors, which mediate contraction, whereas the small coronary arteries are equipped almost exclusively with beta receptors, which mediate relaxation [9]. Considering this fact, it can be surmised that adrenaline may directly (alpha receptors) and indirectly (beta receptors) cause the contraction of the large coronary arteries. 

In some cases, adrenaline may be harmful to patients with Kounis syndrome because it may aggravate coronary vasoconstriction and increase the extent of ischemia, prolong the QT interval, and induce coronary vasospasms and arrhythmias [10]. In these situations, glucagon (the second-line drug for treating anaphylaxis) may be used. Glucagon binds G-coupled surface receptors found throughout the body and, binding to the glucagon receptors activates adenylate cyclase, which in turn raises cAMP levels, resulting in many effects including relaxation of smooth muscle and positive inotropic effects [11-12], for the treatment of anaphylaxis. Therefore, this is another treatment option for anaphylaxis with a different mechanism than adrenaline. However, the administration of adrenaline to patients with Kounis syndrome should be considered, especially for those with severe anaphylaxis.

In the present case, the patient was in danger because of severe anaphylaxis (demonstrating disturbed consciousness and low blood pressure) and glucagon was not available in our ED. Additionally, there are no evidence-based recommendations against intramuscular adrenaline injection in patients with Kounis syndrome. Therefore, adrenaline was administered, which was followed by improvements in the patient’s ECG, echocardiogram, and symptoms; there were no adverse effects. Besides, the changes, over time, of his ECG and echocardiogram findings were crucial for the decision of the clinical management and treatment. The accumulation of such data might also contribute to the realization of individualized medicine for Kounis syndrome.

For cases in which investigations or treatment with the use of radiocontrast media is difficult to perform, such as in this case, we believe that the study about the subtype of Kounis syndrome that does not require treatment like CAG or subsequent percutaneous coronary intervention should also be considered. The data would provide these cases with evidence-based follow-up strategies, which might help us recognize when to intervene while being safe. Besides, in our case, Kounis syndrome was caused by radiocontrast media but the patient’s condition precluded emergent CAG as the allergen could not be easily removed due to his requiring hemodialysis. Thus, the necessity of CAG and/or emergency hemodialysis to remove the allergen for patients with Kounis syndrome requires additional discussion.

Hemodialysis patients can have a higher risk of coronary artery spasms and may be vulnerable to Kounis syndrome. An increase in atherosclerosis risk is also observed in hemodialysis patients and in those with early chronic kidney disease [13]. An animal study reported that atherosclerotic changes may be among the primary factors leading to coronary artery spasms [14]. In a clinical study examining intravascular ultrasound in patients, atherosclerosis was noted at the sites of focal vasospasms, even in the absence of angiographically significant coronary disease [15]. Therefore, patients with chronic kidney disease, including hemodialysis patients, may have a higher risk of coronary artery spasms and, therefore, may be vulnerable to Kounis syndrome.

## Conclusions

We reported a case of Kounis syndrome caused by radiocontrast media in a patient undergoing hemodialysis. We considered that the risk of myocardial ischemia caused by repeated use of radiocontrast media at emergency coronary angiography outweighs the benefits of making a diagnosis. We continued close observation because emergent hemodialysis might have deteriorated the patient’s unstable hemodynamics. The successful treatment of the patient with intramuscular adrenaline raises questions about the perceived necessity of CAG and emergency hemodialysis in these patients. Studies about the subtype of Kounis syndrome that does not need treatment with the use of radiocontrast media should also be considered and would provide these cases with evidence-based follow-up strategies. Thus, more investigations and data about these topics are necessary.
